# Investigating the structural validity and reliability of the sexual health literacy for adults (SHELA) questionnaire among a sample of women in Qazvin, Iran

**DOI:** 10.1186/s12905-022-02112-2

**Published:** 2022-12-16

**Authors:** Rahman Panahi, Leila Dehghankar, Mohiadin Amjadian

**Affiliations:** 1 Research Center for Social Determinants of Health, Research Institute for Endocrine Sciences, Shahid Beheshti University of Medical Sciences, Tehran, Iran; 2 Department of Nursing, Social Determinants of Health Research Center, Research Institute for Prevention of Non-Communicable Diseases, School of Nursing and Midwifery, Qazvin University of Medical Sciences, Qazvin, Iran; 3 Department of English Language, Faculty of Medicine, Kurdistan University of Medical Sciences, Sanandaj, Iran

**Keywords:** Health literacy, Women, Psychometrics, Sexual health

## Abstract

**Background:**

The correct measurement of sexual health literacy requires an instrument with desirable psychometric properties and fitness to the sociocultural context. Despite acceptable psychometric properties of the sexual health literacy for adults questionnaire in the mixed population of men and women, the validity and reliability of this questionnaire in the female population were not determined. Therefore, considering differences in the study population, this study aimed to determine the structural validity and reliability of the questionnaire among women.

**Methods:**

The present study was a methodological and psychometric study of instruments conducted among 310 women referring to healthcare centers in Qazvin, Iran in 2020. Sampling was done using a one-step cluster method. We used Cronbach's alpha coefficient, Pearson correlation coefficient, and confirmatory and exploratory factor analyses to determine the reliability, convergence validity, and construct validity of the questionnaire respectively. Also, the Sexual Quality of Life-Female questionnaire (SQOL-F) and the Female Sexual Function Index (FSFI) were used to evaluate the convergence validity. Data were analyzed using SPSS 20, and STATA 13.

**Results:**

Exploratory factor analysis identified three factors including, “reading and understanding”, “evaluation and application of information”, and “skills of access” which together accounted for 70.85% of the whole variance. Based on the results of confirmatory factor analysis, this questionnaire had overall goodness of fit too. (RMSEA = 0.071, CFI = 0.928, TLI = 0.919, SRMR = 0.041, X2/df = 2.501). Convergent validity of the questionnaire showed a correlation of 0.121–0.243 between the questionnaire's dimensions with the FSFI and the SQOL-F questionnaires respectively. Also, the results showed that the questionnaire had proper internal consistency (Cronbach’s alpha was 0.981) for measuring sexual health literacy in women.

**Conclusions:**

The 39-item sexual health literacy assessment questionnaire consisting of 3 factors in the present study was endowed with sufficient validity and reliability, and it can be used for precisely assessing women’s sexual health literacy.

## Background

Sexual health literacy is a spectrum of literacy in the field of sexual health, which includes various areas such as gender and sexual development, puberty, pregnancy, methods of preventing pregnancy, unwanted pregnancy, sexually transmitted diseases, developing sexual relationship management skills, such as talking about the quality of sexual relations, sexual preferences and compulsions, and the positive and romantic dimensions of sexual relations [1–3]. Sexual health literacy was proposed as a concept that is related to the correct knowledge of sexual health and reproductive health, along with the attitude towards sexual health and reproductive power [4]. Sexual health literacy is a context-based variable and is affected by the ecosystem or a set of cultural and social factors of every society [3, 5]. Acquiring sexual health literacy leads to promoting a correct understanding of duties and responsibilities in sexual relationships, providing the right opportunity for the correct expression of sexual roles, improving the ability to understand and assess the risks related to sexual health, improving individual sexual health, making a safe sexual experience, reducing unwanted pregnancies and sexually transmitted diseases, and improving family and social health [6, 7].

Promoting sexual health literacy requires designing specific social, cultural, and biomedical needs in different communities [8]. One of the main goals of sexual health literacy programs should be empowering people to critically analyze attitudes, beliefs, and cruel methods that prevent them from having freedom in deciding to have safe sex [9].

Assessment of sexual health literacy requires an appropriate instrument [3]. Regarding sexual health literacy, researchers of the present study found three studies [10–12], and only one Iranian questionnaire [3]. This questionnaire was the only native instrument available for measuring sexual health literacy in Iranian adults (SHELA). It was designed and its psychometric properties were determined in the urban population of adults (men and women) by Maasoumi et al. in Tehran. This questionnaire assessed different dimensions of sexual health literacy. The study of Maasoumi et al. [3] showed that it had good content and structure validity, and good reliability in terms of internal correlation.

Women of reproductive age are one of the main bases of fertility in the population; in addition, considering their spousal and maternal roles, they are central to maintaining, securing, and improving family health [13]. Women of productive age make up 22 million of the total population of Iran [14]. Considering the importance of measuring sexual health literacy among women [10], conducting a study in this field requires a specific and standard questionnaire [3]. In other words, the measurement tools and questionnaires that are used in research to measure the achievement of the research goals must be standard to guarantee the obtained results as much as possible [15]. Despite the acceptable psychometric properties of the Sheila questionnaire in a sample of a mixed population (male and female) [3], its validity and reliability in the female population were not clear. Also, the designers of this tool believed that to increase the generalizability of the findings of their study, it was necessary to conduct similar studies using this questionnaire [3]. Therefore, the researchers decided to investigate the construct validity and reliability of this questionnaire among a sample of women.

## Materials and method

### Study design and setting

The present study was a methodological and psychometric study of instruments conducted among 310 women referring to health care centers of Qazvin University of Medical Sciences, Iran in 2020.

### Sampling method and sample size

Sampling was done through a one-stage cluster method so that at first a list of all health centers in Qazvin city was prepared. Then, out of these 24 centers, a center from the north, a center from the south, and a center from the city center were randomly selected and all women referring to these centers, who met the inclusion criteria, were recruited to be part of the study after obtaining written informed consent.

Inclusion criteria included referring to health centers in Qazvin city, having a spouse, having reading and writing literacy, being at least 18 years old, willingness to participate in the study, and having Iranian citizenship. Incomplete completion of the questionnaires and dissatisfaction to go on with the study were considered exclusion criteria.

Moreover, experts recommended a minimum sample size of 5 and a maximum of 20 per item in factor analysis [16, 17]. Thus, considering that the questionnaire had 40 items, 5 people were considered for each item, and the sample size was estimated to be 200 people. However, since the sampling method was a cluster, and also considering 40% of the effect of the study design, the sample size was determined to be about 280 people. Finally, considering the possibility of dropping 10% of the samples, 310 participants entered the study. The exploratory and confirmatory factor analyses were conducted among 305 and 260 women respectively.

### The questionnaires

The data was gathered by a questionnaire that included the followings: (A) demographic and background information including age, level of education, level of education of the spouse, and age of marriage.

(B) Iranian Adult Sexual Health Literacy Assessment Standard Questionnaire (SHELA): This questionnaire included 40 items with four dimensions of accessibility (7 items), reading and comprehension (18 items), evaluation and analysis (5 items), and information application (10 items). The Likert scoring scale included 5 options, with a score of five for strongly agree, four for agree, three for no difference, two for disagree, and one for strongly disagree [3]. To score the questionnaire, first, the raw scores for the four areas of health literacy were calculated, and then they were converted into a standard score between 0 and 100, so that 0 to 50 showed insufficient, 50.1 to 66 less enough, 66.1 to 84 adequate, and scores from 84.1 to 100 excellent health literacies [3].

The validity and reliability of this questionnaire have already been confirmed in the study of Maasoumi et al. [3]; so, the content validity ratio and content validity index of the questionnaire were 0.84 and 0.81 respectively. Also, the results of the exploratory factor analysis indicated the establishment of four factors of access skill, reading and understanding, evaluation and analysis, and information use which shows 68.1% of the total variance. The convergent validity evaluation showed the correlation coefficients between the dimensions of the designed questionnaire and the general health literacy questionnaire were in the range of 0.31–0.7. Also, the internal consistency of the questionnaire with Cronbach’s alpha index for the identified factors was in the range of 0.84–0.94. In addition, the categorical homogeneity of the questionnaire was calculated based on the ICC index and it was in the range of 0.90–0.97 [3].

(C) The Persian version of the Female Sexual Function Index (FSFI) was used to assess women's sexual activity in the last four weeks before the study. This questionnaire included 19 items: sexual desire (2 items, for example: How often did you feel sexual desire or interest over the past four weeks?), arousal (4 items, for example: How often did you feel sexually aroused (“turned on”) during sexual activity or intercourse over the past four weeks?), orgasm (3 items), sexual pain (3 items, for example: when you had sexual stimulation or intercourse, how often did you reach orgasm (climax) over the past four weeks?), genital softening (4 items, for example: How often did you become lubricated (“wet”) during sexual activity or intercourse over the past four weeks?) and sexual satisfaction (3 items, for example: How satisfied have you been with the amount of emotional closeness during sexual activity between you and your partner over the past four weeks?). Each item has 6 choices; ‘I did not have sexual activity = 0’, ‘never = 1’, ‘rarely = 2’, ‘sometimes = 3’, ‘often = 4’ and ‘always = 5.’ The minimum score was 2, the maximum score was 36, and the cut-off point was 28. In other words, scores higher than the cut-off point indicated desirable sexual performance [17].

Also in the study of Panahi et al., Cronbach’s alpha coefficient for FSFI was 0.81. Therefore, the Persian version of FSFI is a reliable tool for assessing the sexual performance of Iranian women [17].

D) To evaluate the quality of women’s sexual life, the Persian version of the Sexual Quality of Life-Female Questionnaire (SQOL-F) was used. This questionnaire was composed of 18 items (Here are three items: When I think about my sexual life, I find it an enjoyable part of my whole life. I have lost my self-confidence as a sexual partner. When I think about my sexual life, I feel like I have lost something) on a six-point Likert scale (strongly agree = 6, agree = 5, neutral = 4, disagree = 5, and strongly disagree = 6). The minimum score was 18 and the maximum was 108. The higher scores indicated a better quality of sex life. To interpret the results, the reference values adopted in that study were classified as follows: (18–36) = poor quality, (37–72) = medium quality, and (73–108) = good quality [17].

This questionnaire was translated and analyzed in 2013. Cronbach’s alpha coefficient was 0.73 and the internal correlation coefficient was 0.88. Also, the content validity index and content validity ratio have been reported to be 0.91 and 0.84, respectively [17]. In the current study, Cronbach’s alpha coefficient for this questionnaire was 0.76.

In fact, two questionnaires (SQOL-F and FSFI) were used to evaluate the convergent validity, because we expected that women who had higher sexual health literacy to have a better quality of sexual life and sexual performance. These two questionnaires and SHELA were given to the studied women at the same time to be completed. Then the correlation between the scores obtained from these three questionnaires was checked using Pearson correlation coefficient.

### Ethical consideration

The Ethical Committee of Qazvin University of Medical Sciences approved the study. The ethics code for this study was IR.QUMS.REC.1399.077.

### Data collection process

At first, issues such as the objectives of the study, having the right to participate freely, having the right to withdraw from the study at any stage according to the individual's request, the confidentiality and anonymity of the questionnaires, and the preservation of the names and addresses of the participating women, were explained to them. Then, written informed consent was obtained from them and the questionnaires were provided to them to be completed.

### Statistical analysis

Data were analyzed using SPSS 20 software (to determine reliability and Pearson correlation coefficient) and STATA 13 (to perform exploratory and confirmatory factor analysis). The reliability coefficient for each scale was calculated using: (a) Cronbach’s alpha, (b) corrected item-total correlations (≥ 0.30), and (c) a value of below 0.10 for the change in Cronbach’s alpha when an item was deleted from the scale. The Kaiser–Meyer–Olkin (KMO) was also used as a measure of sampling adequacy. Principal Component Analysis with Varimax rotation was used to extract the factors (≥ 0.4). Also, the technique of estimation was Robust Maximum Likelihood.

Fit indices were used to examine the fitness of the model, including the ratio of (X2/df < 3) [18], the Comparative Fit Index (CFI > 0.90), Tucker Lewis Index (TLI > 0.95) [19], Root Mean Square Error of Approximation (RMSEA), and Standardized Root Mean Square Residual (SRMSR) in which the values ranged from zero to one [20–22].

## Results

A total of 310 women were entered into the study, of which 5 were excluded (98.4% response rate). However, the number of samples for the exploratory and confirmatory factor analyses in this study did not change and was the same. Of these, 137 (44.9%) were under 30 years old and 168 (55.1%) were over 30 years old. 214 (70.2%) had a university education, 42 (13.8%) had a diploma and 49 (16%) had below diploma degrees. 148 (48.5%) were married under the age of 25 and 157 (51.5%) were over 25. Also, 195 (63.9%) of the spouses had a university, 55 (18.1%) had a diploma and 55 (18%) had below-diploma degrees (Table 1).

**Table 1:** Characteristics of study participants (N = 305)

Characteristics	N (%)
*Age*	
Less than 30 years	137 (44.9%)
Over 30 years	168 (55.1%)
*Education status*	
Below Diploma	16 (16%)
Diploma	42 (13.8%)
Academic	214 (70.2%)
*Age of marriage*	
Less than twenty-five	148 (48.5%)
Over twenty-five	157 (51.5%)
*Education status of the spouses*	
Below Diploma	55 (18%)
Diploma	55 (18.1%)
Academic	195 (63.9%)

### Reliability

Cronbach's alpha coefficient showed that internal consistency and item-total correlation analysis reliability of the scales were acceptable. The total scale coefficients were 0.981 for **SHELA** (Tables 2 and 3).

**Table 2:** Factor loadings, item analysis, and the item total correlations for the 40 items on the SHELA scale (N = 305)

SHELA scale	Factor loading Factor 1	Factor loading Factor 2	Factor loading Factor 3	Item mean (SD)	Corrected item/total correlation	α if item deleted
1. I can get information about sex education in childhood and adolescence from various sources	.349	.368	**.586**	4.21 (.831)	.720	.981
2. I can get information about communicable diseases from different sources	.397	.319	**.641**	4.18 (.880)	.754	.980
3. I can get information about sexual problems and disorders in men and women from different sources	.412	.265	**.731**	4.15 (.868)	.781	.980
4. I can find information about factors affecting sexual relations such as diseases, interpersonal conflicts, marital problems, and complications of the medications taken from various sources	.370	.249	**.742**	(4.08) .946	.753	.980
5. I can obtain information on various methods of pregnancy prevention from various sources	.446	.300	**.616**	4.22 (.900)	.762	.980
6. I can get information about the types of treatment s of Sexual dysfunctions in women and men from different sources	.353	.244	**.785**	4.03 (1.01)	.761	.980
7. I can get information about sex in old age from various sources	.307	.221	**.761**	4.00 (.998)	.706	.981
8. It is easy for me to read about sex education (books, booklets, pamphlets, educational and promotional brochures) during childhood and adolescence	.391	.327	**.567**	4.00 (.972)	.717	.981
9. It is easy for me to read educational materials related to couples' sexual relations and the factors that affect them	**.727**	.274	.409	4.17 (.859)	.822	.980
10. It is easy for me to read educational materials related to sexually transmitted diseases	**.680**	.278	.402	4.15 (.895)	.790	.980
11. It is easy for me to read educational materials related to various methods of contraception	**.744**	.333	.389	4.25 (.828)	.853	.980
12. It is easy for me to read educational materials related to the treatment of sexual dysfunctions in men and women	**.756**	.228	.398	4.15 (.907)	.806	.980
13. It is easy for me to read educational materials related to couples' sexual relations in old age	**.686**	.150	.487	4.09 (.929)	.765	.980
14. If I have a sexual problem and consult a specialist or counselor, it is easy for me to read the written instructions given about my problem	**.751**	.313	.368	4.16 (.883)	.836	.980
15. If I have a sexual problem and refer to a specialist or counselor, it is easy for me to read the guidelines (preparation before tests, pelvic exams, ultrasound, or urogenital examinations)	**.739**	.410	.320	4.21 (.833)	.858	.980
16. I can understand the issues related to the sexual education and training of children and adolescents	**.699**	.294	.366	4.20 (.846)	.790	.980
17. I can understand the issues related to improving the couple's sexual relations	**.766**	.436	.288	4.27 (.822)	.872	.980
18. I understand the issues related to the prevention and treatment of sexually transmitted diseases	**.728**	.440	.318	4.27 (.822)	.867	.980
19. I understand the issues related to improving sexual relations in old age	**.701**	.359	.362	4.13 (.899)	.828	.980
20. In case of sexual problems and referring to a specialist and counselor, I will understand the explanations that she gives me about my problem	**.702**	.463	.270	4.26 (.841)	.835	.980
21. In case of sexual problems and referral, I will understand the meaning and concept of the contents written in the relevant forms such as patient admission form, consent, and file formation	**.756**	.369	.305	4.20 (.852)	.837	.980
22. In case of sexual problems and referral for treatment, I will understand the meaning and concept of the symptoms and the contents written on the signboards in the relevant clinics	**.690**	.412	.333	4.15 (.851)	.835	.980
23. In case of sexual problems and prescribing medicine, I will understand how to use the medicine that is written on the package	**.691**	.450	.273	4.24 (.882)	.824	.980
24. In case of sexual problems and receiving treatment or advice, I will understand its advantages and disadvantages	**.687**	.449	.285	4.24 (.839)	.827	.980
25. I realize the harms of doing things like watching immoral movies, drinking alcohol, smoking, and having extra-marital sex on my sexual health	**.675**	.517	.263	4.31 (.811)	.846	.980
26. I believe in the accuracy of the information I get about sex through various sources	.157	.478	**.574**	3.84 (1.04)	.649	.981
27. I can evaluate the accuracy of sexual health information provided on the Internet	.245	.483	**.599**	3.88 (.970)	.732	.980
28. I can evaluate the accuracy of sexual health information provided by television, radio, and satellite networks	.306	.435	**.580**	3.95 (.981)	.733	.980
29. I can pass on the information I have learned about sexual health to others correctly	.262	.240	.265	4.05 (1.99)	.426	.984
30. As soon as I realize a sexual problem or disorder, I know where or to whom I should go	.388	**.614**	.386	4.03 (.993)	.782	.980
31. If I have a sexual problem, I follow the treatment recommendations such as taking medicine for one hour before sexual intercourse	.237	**.620**	.386	4.05 (.958)	.688	.981
32. In the case of a sexual problem, I will not discontinue the techniques recommended for resolving my sexual problem without the counselor's permission, even if my sexual problem is gone	.336	**.681**	.276	4.08 (.944)	.730	.980
33. If my spouse has a sexual problem, I will go with him for sexual counseling	.356	**.679**	.291	4.14 (.939)	.749	.980
34. Even if I do not have a sexual problem, I go to a sex counselor to get an education and improve the quality of sex with my spouse	.190	**.703**	.267	3.63 (1.13)	.640	.981
35. If I have any questions about my sexual health, I will ask the relevant counselor	.314	**.753**	.296	4.01 (.993)	.769	.980
36. I take care of my sexual health in any situation	.385	**.707**	.289	4.06 (.948)	.779	.980
37. In any situation, I take care of the quality of sex with my spouse	.348	**.744**	.256	4.08 (.979)	.761	.980
38. I avoid asking for sex if my spouse is not physically and mentally ready (sexual coercion)	.438	**.643**	.192	4.13 (.938)	.727	.980
39. When having sex with my spouse, I pay attention to human values such as maintaining dignity, mutual respect, observing moral standards, and so on	.526	**.650**	.215	4.26 (.887)	.800	.980
40. I usually use the information I get from various sources about sexual health	.442	**.661**	.297	4.18 (.871)	.798	.980
Eigenvalue	24.8	1.80	1.69			
Variance (%)	62.10	4.50	4.24			
Total variance (%)	70.85					
Cronbach α	0.981					
Scale mean (SD)	164.66 (29.13)					

Extraction Method: Principal Component Analysis

Rotation Method: Varimax with Kaiser Normalization

Rotation converged in 7 iterations

Bold items with the factor loadings equal or above .500 are significant

### Construct validity

#### Exploratory factor analysis

First, Bartlett's test of Sphericity and KMO measures for sampling adequacy was tested to ensure that the data was suitable for factor analysis. The results showed that the BTS value was significant (df = 780, P < 0.001) and the KMO value was 0.969, suggesting the suitability of the data for analysis. Exploratory factor analysis determined three factors as follows: reading and understanding (9,10,11,12,13,14,15,16,17,18,19,20,21,22,23,24,25), evaluation and application of information (30,31,32,33,34,35,36,37,38,39,40), and skills of access (1,2,3,4,5,6,7,8,26,27,28) respectively with Eigenvalues greater than 1, which together accounted for 70.85% of the variance.

#### Convergent validity of the questionnaire

The questionnaire’s dimensions showed a correlation between 0.121 and 0.243 with the Female Sexual Function Index, and the Sexual Quality of Life-Female questionnaire. Item-total correlation values ranged from 0.39 to 0.74, which justified combining the four-factor model into a three-factor one. The item, “I can pass on the information I have learned about sexual health to others correctly.” did not meet the factor loading criterion, and it was eliminated. Table two summarizes the range of factor loadings for the items in each factor as well as their eigenvalues and variances (Table 2).

#### Confirmatory factor analysis

The results of confirmatory factor analysis with 40 items revealed a good fitting model according to fit indices: X2 = 1648.258, CFI = 0.928, TLI = 0.919, RMSEA = 0.071 RMSR = 0.041 (Table 3).

**Table 3:** Fit indexes of the initial and revised model of the confirmatory factor analyses for SHELA

Indexes values							
	X2	Df	X2/df	CFI	TLI	RMSEA	SRMSR
Initial model	2651.109	699	3.792	0.957	0.949	0.097	0.044
The model	1648.258	659	2.501	0.928	0.919	0.071	

*CFI* comparative fit index; *RMSEA* root mean square error of approximation; *SRMSR* standardized root mean square residual; *TLI* Tucker Lewis index

**Fig. 1:**
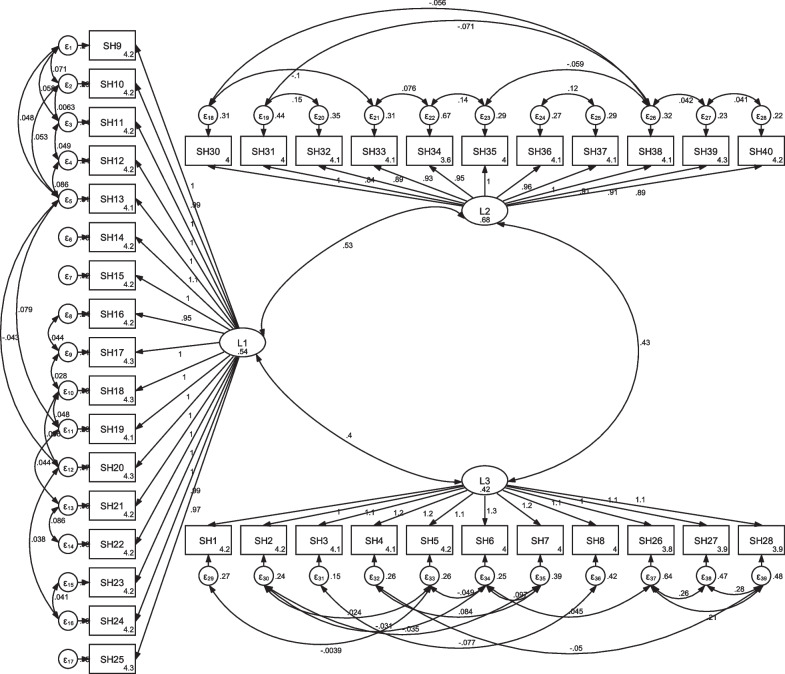
The structure of the questionnaire items and their relationship to their three dimensions

Figure 1 summarized the structure of the questionnaire items and their relationship to their three dimensions. Table 3 showed the fit indices in the confirmatory factor analysis of this questionnaire. Since the SRMSR index was smaller than 0.08 (0.071), the RMSEA index was smaller than 0.1, the X2 / df index was smaller than 5, and the CFI, TLI indices were higher than 0.9, the validity of the instrument was confirmed. Also, Cronbach's alpha coefficients for each dimension and the total questionnaire were high. (Cronbach’s alpha < 0.7) (Table 3).

## Discussion

This study aimed to psychometrically assess the SHELA Questionnaire among women. It provided a sexual adaptation and validation for Iranian women. The findings showed that different factors of the sexual health literacy questionnaire of Iranian adults had a high internal consistency (0.64 to 0.88) that was consistent with the results of the study of Maasoumi et al. [3], in which the Cronbach’s alpha of the items was between 0.84 and 0.94. Also, the overall Cronbach's alpha in the present study was 0.98, which indicated a desirable level of internal consistency of the questionnaire items. This was consistent with the results of the study of Maasoumi et al. [3], in which the overall Cronbach’s alpha was reported to be 0.95. In addition, it is consistent with the results of the study of Panahi et al. [23], in which Cronbach's alpha value of the items was between 0.77 and 0.98. In line with this finding, in the study conducted by Karimi et al. [24] Cronbach’s alpha coefficient of the various factors of the sexual health literacy questionnaire was acceptable.

Moreover, the results of the exploratory factor analysis identified that three factors including “reading and understanding”, “assessment and application of health information” and “skills of access”. In other words, the four-factor model in the original questionnaire was changed into a three-factor one. One of the possible reasons for changing the number of the factors and the items could be the different population and context here; the population was mixed (both men and women) in the main study [3], but it was only women in the present study. Also, three factors of “obtain and access”, “comprehending and understanding” and “decision/behavior” were the main foundations of health literacy definitions. In other definitions, while maintaining this backbone, other dimensions such as “evaluation and judgment” were also added to the definition [25]. Also, in most of the definitions of health literacy, the “reading” skill was embedded in the “obtaining and access” factor [25]. Therefore, the three-factor model of sexual health literacy in the present study could be logical and acceptable. It can be said that measuring health literacy requires different tools in various situations, and since it is not possible to provide a single definition for health literacy, it is also not possible to measure it with a single tool. A few analyses of the structure of the tools designed to measure sexual health literacy in Iran showed different patterns. For example, the tool developed by Dabiri et al. suggested a four-factor structure (reading and understanding, access, information evaluation, decision-making, and information application) to measure sexual health literacy [12]; While the tool designed by Karimi et al. [24]. introduced a structure of seven factors (access, reading, understanding, evaluation, use, communication, and empowerment) to measure sexual health literacy. Meanwhile, Rakhshaee et al. identified five dimensions of sexual health including information needs, information search, information perception, information validation, and information application as the most important aspects of sexual health literacy among women [26]. On the other hand, the results of exploratory factor analysis showed that one item from “the evaluation and analysis” factor (I can properly transfer my learned knowledge about sexual health to others) was omitted due to insufficient factor loading (item number 29) because it was largely associated with communication skills which was a separate area of health literacy [27]. Therefore, its removal seemed logical. Also, the results of exploratory factor analysis showed that one item from “the assessment and analysis” factor (as soon as I realized the problem of sexual impairment, I know where or to whom I should refer) was loaded on the “the application of health information” factor which was changed into “the assessment and application of health information” in the present study. The respondents possibly interpreted this item as doing a behavior; therefore, it was loaded on the “application of health information” factor here. In addition, the results of exploratory factor analysis showed that three items from “the evaluation and analysis” factor were loaded on the “the skills of access” factor. It can be said that having words like information and resources of information such as Internet, TV, and satellite in these items led the respondents to probably interpret them as in the scope of “the skills of access” factor. The results of exploratory factor analysis also showed that an item in the list of “the reading and understanding” factor was loaded on the "the skills of access” factor. It can be said that in most health literacy definitions, reading skills were embedded in the “gaining and access” skill [25]. In the previous study, the number of items related to “the skill of access” was 7; while in the present study, the number of items for this skill is eleven. We know that accessibility skills are the most significant dimension in assessing sexual health literacy [3]. We also know that people must access specific resources and databases to be sexually literate. Rakhshaee et al. showed that providing “the information needs” for women's sexual health was the most important dimension of sexual health literacy for them [26]; therefore, considering the low number of these sources and databases for women in Iran [3], this finding indicated the high need for women to increase access to sexual health information. In general, the present study examined and confirmed the variability of the factors of this questionnaire in the female population and showed that the factors could be variable if used in different populations. Therefore, similar studies are still needed.

Moreover, the findings of the convergent validity test showed that there was a relatively good level of convergence between the SHELA questionnaire and the subscales of two questionnaire FSFI and SQOL-F. In line with the present study, in Maasoumi et al.’s [3] study, there was a favorable level of convergent validity between the SHELA questionnaire and the subscales of the Health Literacy for Iranian Adults questionnaire (HELIA).

The goodness of fit of the Indices of the SHELA questionnaire showed that this 3-factor model with 39 items had a good fit. These findings also confirmed the results of the study of Maasoumi et al. [3]. In addition, the results of confirmatory factor analysis confirmed the construct validity of the questionnaire. The values of the standardized parameters determined the strength of the factor loading of each item on its subscales, and it also determined how much of the total variance was explained by the subscale variance. The larger the factor load, the more variance it explained; in fact, these factor loads determined the total variance of each subscale [28]. Therefore, the factors’ items were appropriately chosen and they could properly evaluate the three hidden variables of the questionnaire including “access”, “reading and understanding”, and “evaluating and using health information”. The results of Karimi et al.’s [24] confirmatory factor analysis showed a favorable construct validity for this questionnaire, which is consistent with our results.

To the best of our knowledge, the present study was the first to examine the psychometric properties of the questionnaire by factor analysis in women. Failure to use other structural validity methods along with exploratory and confirmation factor analysis was one of the most important limitations of the present study because it could increase the validity of the tool used. During designing of SHELA, HELIA questionnaire was used to check the convergence validity. Therefore, not using the HELIA questionnaire to check the convergence validity and compare it with its convergence validity in the previous study, can be another limitation of this study. Also, not using other methods of reliability such as the test-test method was another limitation of the present study. Additionally, the relatively small sample size and the probability of sample bias were other limitations of the present study. In the present study, about 64% of the samples had a university degree which affected the level of health literacy of the participants, and it might be a confusing factor in their responses. Therefore, it is recommended to conduct this study among women with different levels of education. Furthermore, since the study was conducted only among women in several health centers, the results might not be generalizable to other populations. Accordingly, further research on a larger scale is recommended on women, especially in rural areas. It is also suggested to replicate this study among male populations.

## Conclusion

This is the first study that tested the psychometric properties of the sexual health literacy for adults (SHELA) questionnaire in Iranian women and provided a cross-sexual adaptation and validation for this questionnaire. Also, comprehensive psychometric work will help provide suitable measures of sexual health literacy that capture the distinct components of this concept in women properly. Therefore, the results showed that the 39-item Sexual Health Literacy Assessment Questionnaire with three factors was endowed with sufficient validity and reliability and it can be useful to precisely assess Iranian women’s sexual health literacy.

## Data Availability

The datasets used and/or analyzed during the current study are available from the corresponding author upon reasonable request.

## Abbreviations

SHELA: Sexual health literacy for adults
FSFI: Female sexual function index
SQOL-F: Sexual quality of life-female questionnaire
HELIA: Health Literacy for Iranian Adults
IR.QUMS.REC: Iran. Qazvin University of Medical Sciences. Recording
KMO: Kaiser–Meyer–Olkin
CFI: Comparative fit index
CFA: Component factor analysis
RMSEA: Root mean square error of approximation
SRMSR: Standardized root mean square residual
CVI: Content validity indices
BTS: Bartlett test of sphericity
